# Phytochemicals of *Minthostachys diffusa* Epling and Their Health-Promoting Bioactivities

**DOI:** 10.3390/foods9020144

**Published:** 2020-02-01

**Authors:** Immacolata Faraone, Daniela Russo, Lucia Chiummiento, Eloy Fernandez, Alka Choudhary, Magnus Monné, Luigi Milella, Dilip K. Rai

**Affiliations:** 1 Department of Science, University of Basilicata, V.le dell’Ateneo Lucano, 10, 85100 Potenza, Italy; immafaraone88@gmail.com (I.F.); daniela.russo@unibas.it (D.R.); lucia.chiummiento@unibas.it (L.C.); magnus.monne@unibas.it (M.M.); 2 Spinoff BioActiPlant s.r.l., Università della Basilicata, V.le dell’Ateneo Lucano, 10, 85100 Potenza, Italy; 3 Department of Crop Sciences and Agroforestry, Faculty of Tropical AgriSciences, Czech University of Life Sciences, Praha 6-Suchdol, Kamýcká 129, 16500 Prague, Czech Republic; eloy@ftz.czu.cz; 4 Department of Food BioSciences, Teagasc Food Research Centre Ashtown, D15KN3K Dublin, Ireland; alkachoudhary12@gmail.com (A.C.); dilip.rai@teagasc.ie (D.K.R.)

**Keywords:** *Minthostachys diffusa*, Lamiaceae, DPPH, *beta*-carotene bleaching, relative antioxidant capacity index (RACI), polyphenols, terpenoids, liquid chromatography–mass spectrometry analysis (UHPLC-MS/MS), flavonoids

## Abstract

The genus *Minthostachys* belonging to the Lamiaceae family, and is an important South American mint genus used commonly in folk medicine as an aroma in cooking. The phytochemical-rich samples of the aerial parts of *Minthostachys diffusa* Epling. were tested for pharmacological and health-promoting bioactivities using in vitro chemical and enzymatic assays. A range of radical scavenging activities of the samples against biological radicals such as nitric oxide and superoxide anion and against synthetic 2,2-diphenyl-1-picrylhydrazyl and 2,2′-azino-bis(3-ethylbenzothiazoline-6-sulfonic acid) radicals, the ferric reducing antioxidant power and the lipid peroxidation inhibition were determined and ranked using the ‘relative antioxidant capacity index’ (RACI). The ethyl acetate fraction showed the highest RACI of +1.12. Analysis of the various fractions’ inhibitory ability against enzymes involved in diabetes (α-amylase and α-glucosidase), and against enzymes associated with Parkinson’s or Alzheimer’s diseases (acetylcholinesterase and butyrylcholinesterase) also suggested that the ethyl acetate fraction was the most active. Liquid chromatography–tandem mass spectrometry analysis of the ethyl acetate fraction showed more than 30 polyphenolic compounds, including triterpenes. The inhibitory cholinesterase effects of the triterpenes identified from *M. diffusa* were further analysed by in silico docking of these compounds into 3D-structures of acetylcholinesterase and butyrylcholinesterase. This is the first study on pharmacological activities and phytochemical profiling of the aerial parts of *M. diffusa*, showing that this plant, normally used as food in South America, is also rich in health-promoting phytochemicals.

## 1. Introduction

Plant metabolism produces numerous secondary metabolites (phytochemicals) that are very specific to each plant family and do not participate directly in the growth and development of the plant and [1,2]. Phytochemicals are known to possess a wide range of properties including antioxidant, hypoglycaemic, anticholinesterase, hypolipidemic, antiviral, antibacterial, antifungal, anti-inflammatory and cytotoxic activities as comprehensively reviewed by Pinakin et al. 2020 and Tang et al. 2019 [3,4]. Although plants are considered an important natural source for therapeutic applications with well-known ethnomedical uses in literature, yet they have been poorly investigated from the phytochemical point of view as exemplified by a recent review on the genus *Tragopogon* of Asteraceae family [5]. The plants belonging to *Minthostachys* genus are also among the less studied. The local populations call them “*peperina*” in Argentina and “*mu*ñ*a*” in the area from central Peru to Bolivia.

From the early 16th century the folklore medicinal use of the *Minthostachys* genus has been reported for the treatment of several health-disorders such as headache, cold and flu, respiratory illnesses (asthma, bronchitis, cough), digestive disorders (indigestion, carminative, stomach-ache, diarrhea, colics), muscle spasms, rheumatism, impotence and amenorrhea [6,7]. Other traditional uses of *Minthostachys* include biopesticides (antimycotic and antiparasitic, against flea infestations) and for the protection of stored potato and oca tubers from aphids and pests [6,7]. In recent years, there have been numerous research studies on *Minthostachys* oils to provide scientific evidences on their medicinal properties [8,9,10,11]. For instance, the main components of *Minthostachys verticillata* (*M. verticillata*) essential oil, namely pulegone (63.4%), menthone (15.9%), and limonene (2.1%), have been linked immediate-type allergic reactions in vitro and in vivo [8]. Montironi et al. (2016) have shown the bactericidal efficacy of *M. verticillata* essential oil against *Streptococcus uberis* strains isolated from bovine mastitis [9], while the essential oil of *Minthostachys mollis* (*M. mollis*) that largely contained pulegone (55.2%) and trans-menthone (31.5%) showed significant efficacy against Gram-positive and Gram-negative bacteria, especially *Bacillus subtilis* and *Salmonella typhi*, at 4 μg/mL [10]. A natural product, (−)-(1*S*,2*R*,3*R*,4*S*)-1,2-epoxy-1-methyl-4-(1-methylethyl)-cyclohex-3-yl acetate, isolated from the volatile constituents of *Minthostachys tomentosa* exhibited significant insecticidal activity against *Oncopeltus fasciatus*, whereas its synthetic form was found inactive [11]. However, there is no report on the effects of *Minthostachys* on chronic degenerative diseases such as diabetes and Alzheimer’s diseases that are associated with oxidative stress. According to the World Health Organization, there are nearly 422 million people worldwide with diabetes, which is one of the major causes of death globally (https://www.who.int/health-topics/diabetes). An increasing number of studies have also linked diabetes with neurodegeneration, which, for example, is involved in the development of Alzheimer’s disease [12,13,14].

Although the *Minthostachys* genus has received growing attention from modern pharmacology and medicine, the interest in this genus has been concentrated only on few species, mainly *M. verticillata, M. mollis, Minthostachys andina* or *Minthostachys glabrescens*, whereas very few studies have been focused on *Minthostachys diffusa* (*M. diffusa*) Epling [6,7,8,9,10,11]. *M. diffusa* is also known as “*tusuwaya*”, an endemic species prevalent in Bolivia, where the local population uses its aerial part as tea and to treat digestive, spasms and carminative disorders [6], but its properties and phytochemical composition have not been explored yet.

In this study, the aerial parts of *M. diffusa* powders were subjected to sequential extraction using solvents of different polarities. All the samples were tested for their antioxidant activity with different in vitro methods. Furthermore, in vitro assays have been used to assess the sample inhibitory activities on enzymes involved in diabetes (i.e., α-amylase and α-glucosidase) and neurodegenerative diseases (acetylcholinesterase and butyrylcholinesterase). Spectrophotometric and liquid chromatography–tandem mass spectrometry (LC-MS/MS) methods were performed on the most active samples to characterize and quantify the secondary metabolites responsible for their biological activities. In silico docking analysis to confirm the inhibitory effects of the identified compounds against cholinesterase enzymes was also carried out. To the best of our knowledge, this is the first study on the potential pharmacological activity as antioxidant, antidiabetic, and anticholinesterase of *M. diffusa*, besides its phytochemical characterization by LC-MS/MS analysis.

## 2. Materials and Methods

### 2.1. Chemicals

Solvents such as chloroform, ethanol, ethyl acetate, glacial acetic acid, hydrochloric acid, methanol, *n*-butanol, *n*-hexane, and phosphoric acid were purchased from Carlo Erba (Milan, Italy). Acetonitrile, formic acid and Leucine-Enkephalin were purchased from Merck (Wicklow, Ireland). Folin-Ciocalteu reagent, sodium carbonate, 2,2-diphenyl-1-picrylhydrazyl (DPPH), 2,2′-azino-bis(3-ethylbenzothiazoline-6-sulfonic acid) (ABTS), potassium persulfate, β-nicotinamide adenine dinucleotide reduced form (NADH), phenazine methosulfate (PMS), nitrotetrazolium blue chloride (NBT), sodium nitroprusside dehydrate (SNP), sulfanilamide, *N*-(1-naphthyl)ethylenediamine dihydrochloride, sodium acetate trihydrate, 2,4,6-tripyridyl-*s*-triazine, 3,5-dinitrosalicylic acid, 4-*p*-nitrophenyl-α-d-glucopyranoside, 5,5′-dithio-bis(2-nitrobenzoic acid), α*-*amylase from hog pancreas (CAS number: 9000-90-2), α-glucosidase from *Saccharomyces cerevisiae* (CAS number: 9001-42-7), acetylcholinesterase (AChE) from *Electrophorus electricus* (electric eel, type VI-s, lyophilized powder), acetylthiocholine iodide, β-carotene, bovine serum albumin, butyrylcholinesterase (BChE) from equine serum (lyophilized powder), iron (III) chloride (FeCl_3_.6H_2_O), linoleic acid, potassium phosphate monobasic, potassium sodium tartrate tetrahydrate, *s*-butyrylthiocholine chloride, sodium chloride, sodium hydroxide, sodium phosphate, starch, trizma hydrochloride, aluminium chloride, galantamine, 6-hydroxy-2,5,7,8-tetramethylchroman-2-carboxylic acid (Trolox), acarbose, butylhydroxytoluene (BHT), gallic acid, quercetin, linalool and Tween 20 were purchased from Sigma-Aldrich (Milan, Italy). Polyphenol standards for LC-MS/MS were purchased either from Extrasynthese (Genay, France) or from Merck (Wicklow, Ireland). Milli-Q water was obtained from Mill-Q purification system (Millipore, Bedford, MA, USA).

### 2.2. Plant Material and Samples Preparation

The aerial parts of *M. diffusa* (Md) were collected near the Aymaya community (18°26′54″ S to 66°27′36″ W; 3750 msnm), Bustillo province, Potosí department, Bolivia, in 2014. A voucher specimen was stored at the National University Siglo XX, Llallagua, Potosí, Bolivia.

The aerial parts were dried at room temperature. Briefly, 95 g of dried plant material were crushed and subjected to exhaustive dynamic maceration in a shaker set at 25 °C with 96% ethanol (Md EtOH) for 24 h. Four extractions at a solid to solvent ratio of 1:15 (*w*/*v*) per extraction were performed. The four Md EtOH extracts from each plant material were combined and filtered through a Buchner funnel (0.45 µm) and dried. Then, a part of this extract (7 g in 100 mL of water) was subjected to liquid/liquid extraction in triplicate using *n*-hexane, chloroform, ethyl acetate and *n*-butanol in order to separate the compounds on the basis of their increasing solvent polarity [15,16,17]. All fractions (*n*-hexane (MdH), chloroform (MdC), ethyl acetate (MdEA), *n*-butanol (MdB) and water (MdW)) were dried and stored in darkness at room temperature until further experimental use. The extraction yield was determined as follows:

Extraction yield of Md EtOH (% *w*/*w*) = [total obtained dried extract (gram)/initial dried plant materials (gram)] × 100;

Extraction yields of fractions (% *w*/*w*) = (single obtained dried fraction (gram)/initial dried Md EtOH subjected to liquid/liquid extraction (gram)) × 100.

### 2.3. Total Phenolic, Flavonoid and Terpenoid Contents

The Folin–Ciocalteu reagent was used to determine the total phenolic content present in the analysed samples using a colorimetric assay by adapting the method of Singelton et al. [18]. A calibration curve with gallic acid as a standard was made and the results were expressed as ‘milligrams of gallic acid equivalents per gram’ (mg GAE/g) of dried sample.

Total flavonoid content was determined using aluminum chloride as the reactant reagent and quercetin to obtain the standard curve [19]. The results were expressed as ‘milligrams of Quercetin Equivalents per gram’ (mg QE/g) of dried sample. Monoterpene linalool was used as standard reagent for the determination of total terpenoids content as described previously [20]. The results were expressed as ‘milligrams of Linalool Equivalents per gram’ (mg LE/g) of dried sample.

### 2.4. Antioxidant Activity

#### 2.4.1. Radical Scavenging Activity

All *M. diffusa* samples were tested for their radical scavenging activity by four different in vitro chemical assays targeted against the biological super oxide anion (O_2_^−^) and nitric oxide (NO) radicals, and the synthetic neutral DPPH and cationic ABTS^+^ radicals [21]. The ability of the various samples to scavenge the radicals was monitored spectrophotometrically and the results were expressed as the concentration (in mg/mL) inhibiting 25% of radicals (IC_25_) or quantified in ‘milligrams of Trolox Equivalents per gram’ (mg TE/g) of dried sample.

#### 2.4.2. Ferric Reducing Antioxidant Power Assay (FRAP)

The ability of samples to reduce the Fe (III) in Fe (II) was monitored in FRAP assay at 593 nm [22]. The Trolox was used as a standard and FRAP values were expressed as mg TE/g.

#### 2.4.3. β-carotene Bleaching Assay

The capacity of samples at final concentration of 0.05 mg/mL to inhibit the lipid peroxidation was evaluated in the β-carotene emulsion at 470 nm [14]. BHT was used as positive control and the results were expressed as percentage of β-carotene bleaching inhibition (% Antioxidant Activity (%AA)) [15].

#### 2.4.4. Relative Antioxidant Capacity Index (RACI)

No single chemical test can define a complete antioxidant capacity of a sample. For this reason, it is necessary to perform more than one in vitro antioxidant assay. However, the measurement scale of antioxidant of each method is different, which makes difficult to define the antioxidant capacity of the sample. Here a statistical method RACI that integrates the results obtained from different in vitro antioxidant assays was used. RACI is an arbitrary index which allows to rank the antioxidant capacity derived from different antioxidant methods.

RACI is derived by comparing the mean and the standard deviation of the raw data of each antioxidant method. The standard score represents the distance between the raw data and the mean in units of the standard deviation, which is negative when the raw data are smaller than the mean and vice-versa. The final data of RACI were represented in a histogram similar to previously described publications [15,17].

### 2.5. Potential Antidiabetic Activity

#### 2.5.1. α-amylase Inhibition

The α-amylase enzyme from hog pancreas was mixed with different concentrations of each sample and starch used as substrates [23]. Briefly, the aromatic yellow-orange 3,5-dinitrosalicylic acid reagent was added that reacts with reducing sugars released from starch hydrolysis and other reducing molecules to form 3-amino-5-nitrosalicylic acid, which was subsequently monitored at 540 nm. The clinical antidiabetic drug, acarbose, was used as a positive control and the results were expressed as ‘milligrams of acarbose equivalents per gram’ (mg AE/g) of dried sample or as the concentration (in mg/mL) of the sample required to inhibit the activity of the enzyme by 50% (IC_50_) calculated by non-linear regression analysis.

#### 2.5.2. α-glucosidase Inhibition

The inhibition of α-glucosidase enzyme was performed as previously described [23]. The α-glucosidase enzyme, the substrate 4-*p*-nitrophenyl-α-d-glucopyranoside, and different concentrations of each sample were mixed, and the reaction was spectrophotometrically monitored at 405 nm by the release of yellow *p*-nitrophenol. In this assay also, acarbose was used as a positive control and the results were expressed as mg AE/g or IC_50_.

### 2.6. Anticholinesterase Activity

The two prevalent forms of cholinesterase in a healthy brain are acetylcholinesterase (AChE) and butyrylcholinesterase (BChE). Ellman’s reaction to analyse the AChE and BChE inhibition by the samples was used as described before [17]. The natural drug galantamine, commonly used in the treatment of Parkinson’s and Alzheimer’s diseases, was used as a positive control and the results were expressed as ‘milligrams of galantamine equivalents per gram’ (mg GE/g) of dried sample or IC_50_.

### 2.7. Liquid Chromatography Tandem Mass Spectrometry Analysis of Polyphenols

The selected samples based on the highest biological activities, in particular ethyl acetate and *n*-hexane fractions of *M. diffusa*, were chosen for structural characterization of polyphenols on a Q-Tof Premier mass spectrometer coupled to an Alliance 2695 HPLC system (Waters Corporation, Milford, MA, USA). The quantification of identified polyphenols was performed using a Waters Acquity ultra-high-performance liquid chromatography tandem mass spectrometry (UHPLC-MS/MS), as described previously [15,17].

### 2.8. Molecular Docking

Structural homology models of *Electrophorus electricus* AChE and equine BChE, which were used in the in vitro inhibition assays, were generated by Swiss-model based on the structures of *Tetronarce californica* AChE (PDBID: 1GQS, with 70% sequence identity with *E. electricus* AChE) and *Homo sapiens* BChE (PDBID: 5LKR, with 91% sequence identity with equine BChE), respectively [24]. The resulting models were further refined by YASARA energy minimization [25]. In silico molecular docking of conformationally flexible terpenes identified in *M. diffusa* into semi-rigid homology models of AChE and BChE was performed with AutoDock Vina [26].

### 2.9. Statistical Analysis

All analysis and assays were performed in triplicates and the data were expressed as mean ± standard deviation. The correlation among used assays was verified by the calculation of *p* value by one-way analysis of variance (ANOVA) using GraphPad Prism 5 Software (San Diego, CA, USA). Only the *p* ≤ 0.05 was considered significant.

## 3. Results and Discussion

### 3.1. Extraction Yield and Influence of Solvents on Total Polyphenolic, Flavonoid and Terpenoid Contents

The exhaustive extraction of aerial parts of *M. diffusa* in the 96% ethanol showed a yield of 12.30 ± 1.07%. Previous studies on other species of the *Minthostachys* genus have reported varying extraction yields. For example, extraction yield of ethanolic extracts of the aerial parts of *M. verticillata* was 3.60% [27], which is considerably lower than the values obtained in our study. On the other hand, the infusions of *M. mollis* and *M. verticillata* yielded a high extraction yield of 20.80% [28,29].

**Figure 1:**
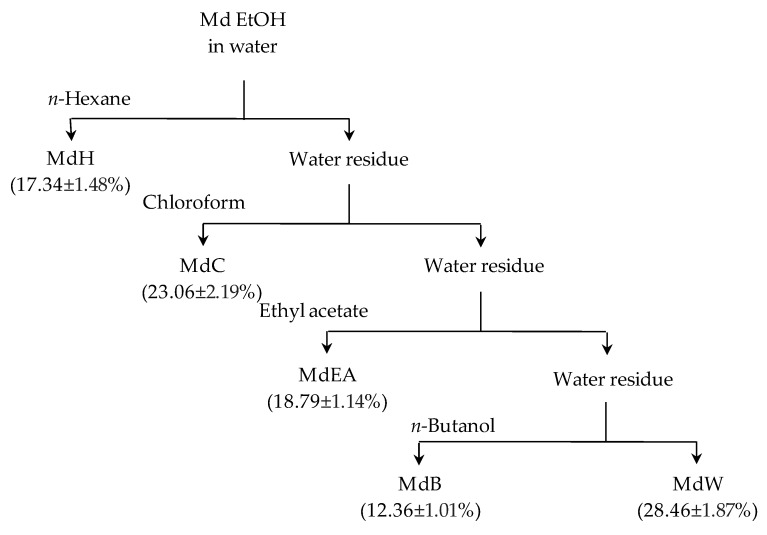
Extraction yields (%) of *M. diffusa* EtOH extract partitioned fractions with solvents of different polarities. Results are expressed as mean ± standard deviation of the triplicate experiments. Samples are crude ethanol extract (Md EtOH), *n*-hexane fraction (MdH), chloroform fraction (MdC), ethyl acetate fraction (MdEA), *n*-butanol fraction (MdB) and water fraction (MdW).

The extraction yields following the sequential liquid/liquid partitioning of ethanolic extract of *M. diffusa* (Md EtOH) in various solvents of different polarities are shown in Figure 1. The fractions that showed the highest extraction yields were water (MdW) and chloroform (MdC) fractions (28.46 ± 1.87% and 23.06 ± 2.19%, respectively). The butanol fraction (MdB) showed the lowest extraction yield (12.36 ± 1.01%).

**Figure 2:**
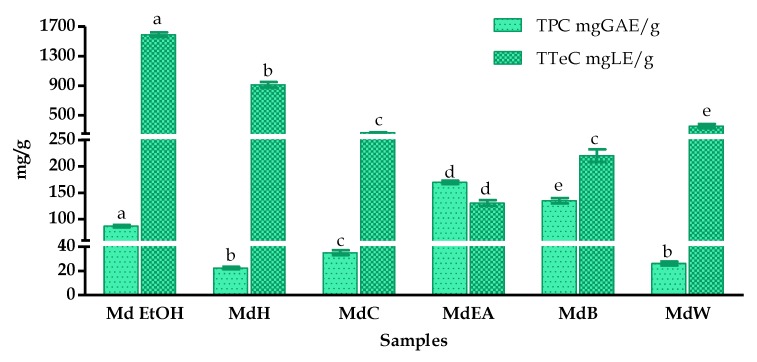
Total Polyphenol Content (TPC) and Total Terpenoid Content (TTeC) of *M. diffusa* fractionated samples. Results are expressed as mean ± standard deviation of triplicate determinations in ‘mg of Gallic Acid Equivalents per gram’ (mg GAE/g) of dried sample and in ‘mg of Linalool Equivalents per gram’ (mg LE/g) of dried sample. In each test, the values with the same letter (a, b, c, d, e) are not significantly different at the 95% confidence limit according to one-way analysis of variance (ANOVA). Samples are crude ethanol extract (Md EtOH), *n*-hexane fraction (MdH), chloroform fraction (MdC), ethyl acetate fraction (MdEA), *n*-butanol fraction (MdB) and water fraction (MdW).

The samples from various partitioned fractions showed statistically significant differences in total phenolic content (TPC) and total terpenoid content (TTeC) as illustrated graphically in Figure 2. The mean TPC value of all fractions was 79.29 mg of GAE/g, where the MdEA and MdB fractions showed higher TPC values (i.e., 169.74 ± 3.10 and 135.11 ± 5.22 mg GAE/g, respectively) than other fractions. Similarly, the Md EtOH extract showed the highest TTeC (1590.31 ± 32.33 mg LE/g), which was significantly higher than the mean value of 577.80 mg LE/g. The total flavonoid content (TFC) assay was carried out only on *M. diffusa* fractions that presented a TPC value higher than the mean value, i.e., the Md EtOH, MdEA and MdB fractions. The Md EtOH fraction showed the highest TFC value (400.84 ± 26.94 mg QE/g) followed by MdEA and MdB (177.33 ± 14.05 and 114.23 ± 6.03 mg QE/g, respectively) (data not shown). Based on these findings, the phenolic, flavonoid, and terpenoid contents depend on the choice of solvents used for the extraction due to differences in the chemistry of these classes of compounds.

### 3.2. Antioxidant Activity

Six complementary in vitro antioxidant assays were performed to determine the antioxidant activity of *M. diffusa* samples. The ability of samples to scavenge the biological superoxide anion (O_2_^−^) and nitric oxide (^.^NO) radicals was expressed as IC_25_ and results were compared with the ascorbic acid values.

All six samples caused a dose-dependent inhibition of superoxide anion. The butanol (MdB) fraction showed an IC_25_ of 0.26 ± 0.01 mg/mL that was very similar to that of ascorbic acid (IC_25_ of 0.26 ± 0.02 mg/mL). MdW and MdC fractions presented the lowest scavenging activity. This also corresponded with the biological nitric oxide scavenging assay, where the activity was detectable only in the MdB fraction (IC_25_ of 2.50 ± 0.15 mg/mL), which was better than the ascorbic acid (IC_25_ of 4.78 ± 0.09 mg/mL). On the contrary, the ethyl acetate (MdEA) fraction showed the highest radical scavenging-activity against synthetic radicals (Table 1) with 444.76 ± 28.24 mg TE/g and 281.64 ± 7.93 mg TE/g in ABTS and DPPH, respectively, followed by MdB (218.56 ± 9.38 and 122.30 ± 2.77 mg TE/g in ABTS and DPPH, respectively). The MdC and MdW fractions showed the lowest scavenging activity against the synthetic radicals. A similar trend was observed for the ferric reducing antioxidant power (574.86 ± 9.14 and 297.75 ± 5.71 mg TE/g for MdEA and MdB, respectively), while the MdC and MdH fractions were the least active. The inhibition of the lipid peroxidation determined by β-carotene bleaching (BCB) test showed the most active sample was the MdEA fraction (19.13 ± 0.93% AA), whereas the MdB and MdW fractions did not show any activity.

The correlation between the amount of polyphenols and the antioxidant activity of the fractions was evaluated by the Pearson correlation coefficient (Table 2). The highest correlation was observed between the total polyphenol content and radical-scavenging activity (r_TPC/ABTS_ = 0.96 and r_TPC/DPPH_ = 0.94) or ferric reducing power (r_TPC/FRAP_ = 0.96). The ferric reducing power and the radical-scavenging activity against ABTS and DPPH radicals (r_FRAP/ABTS_ = 1.00 and r_FRAP/DPPH_ = 0.99) also displayed high correlation constants. There were also very good correlations between ABTS and DPPH assays (r_ABTS/DPPH_ = 0.99) and between NO and SO tests (r_NO/SO_ = 0.91). The terpenoids were poorly correlated with the antioxidant activities (*r* < 0 for terpenoids against all assays except for BCB test r_TTeC/BCB_ = 0.06).

**Figure 3:**
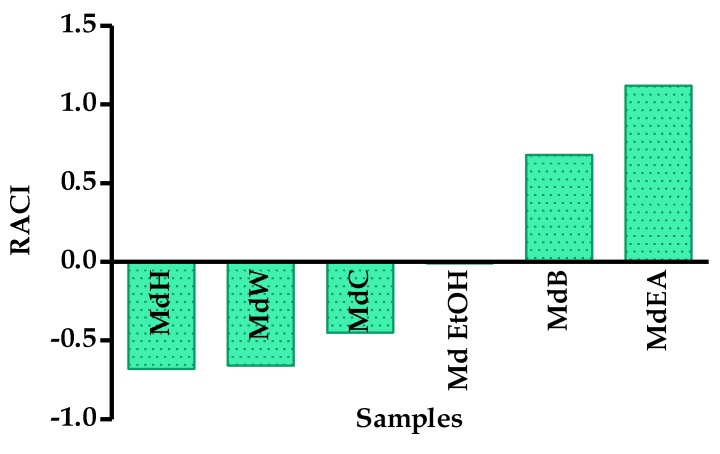
Relative Antioxidant Capacity Index (RACI) of different *M. diffusa* samples.

The integration of obtained results by the six different in vitro antioxidant assays was calculated through the relative antioxidant capacity index (RACI) in order to compare and rank the data (Figure 3). The MdEA fraction showed the highest RACI value (+1.12) followed by the MdB fraction (0.68). The MdW and MdH fractions presented the negative RACI (−0.66 and −0.68, respectively) implying a relative lack of antioxidant activity by these fractions.

To the best of our knowledge, this is the first study of antioxidant activity of aerial parts of *M. diffusa*. However, studies on other species belonging to the *Minthostachys* genus are reported in the literature. The infusion extract of *M. verticillata* (2 g in 250 mL of boiling water) has been reported to have a low radical scavenging activity against the neutral DPPH due to its low phenolic content [28]. Interestingly, the essential oil obtained from leaves of *M. spicata* by hydrodistillation exhibited a higher DPPH radical scavenging efficacy (76.05 ± 2.40%) at 500 μg/mL and had an IC_50_ value of 82.19 ± 6.70 μg/mL, which may be compared to our results where the Md EtOH presented an IC_50_ value of 269.45 ± 8.24 μg/mL. The high activity in *M. spicata* was attributed to the major oil constituents (pulegone, isomenthone, and menthone) as well as oxygenated monoterpenes in general [29]. Nevertheless, the MdEA fraction, the most active sample of *M. diffusa*, showed an IC_50_ value of 108.14 ± 3.07 μg/mL after 30 min of incubation and IC_50_ of 90.04 ± 3.44 μg/mL after 90 min, which was close to that of essential oils from *M. spicata* leaves.

### 3.3. Potential Antidiabetic Activity

**Figure 4:**
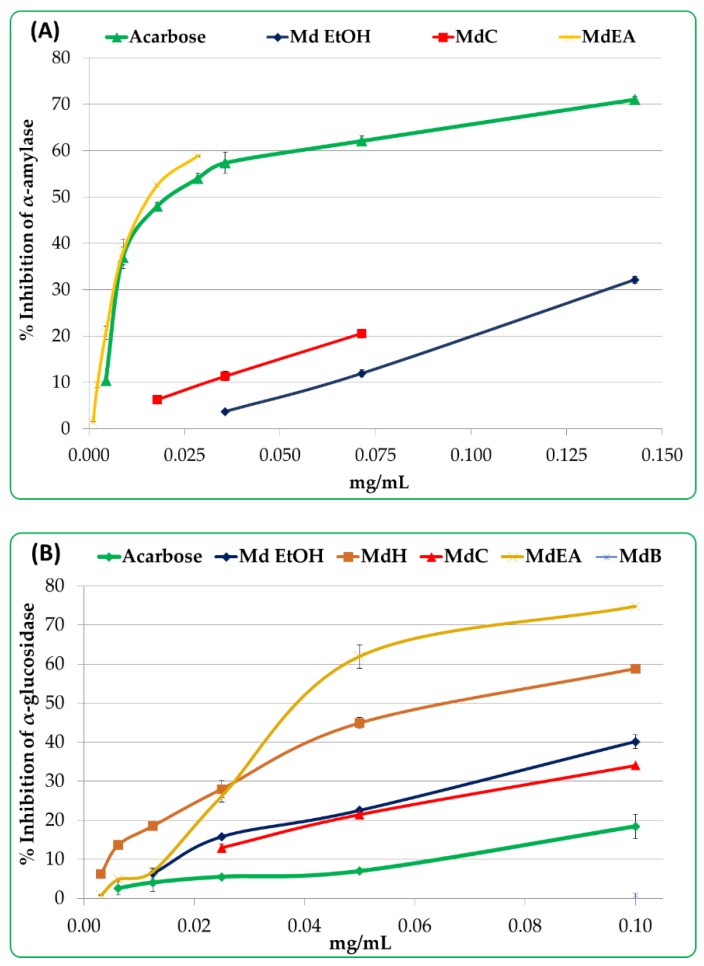
α-amylase (**A**) and α-glucosidase (**B**) inhibition activity of *M. diffusa* samples. Samples are acarbose, crude ethanol extract (Md EtOH), *n*-hexane fraction (MdH), chloroform fraction (MdC), ethyl acetate fraction (MdEA) and *n*-butanol fraction (MdB).

The capacity of the *M. diffusa* samples to inhibit enzymes, namely α-amylase and α-glucosidase that are involved in the pathogenesis of diabetes, were tested and the results show a concentration-dependent inhibition (Figure 4). The ethyl acetate fraction (MdEA) showed promising inhibition ability against α-amylase with an IC_50_ value of 16.40 ± 1.61 μg/mL. The MdH and MdB fractions did not inhibit α-amylase, while the MdW fraction had no effect in any of the two assays. In the α-glucosidase inhibition assay, IC_50_ for the MdB and MdW fractions was not reached in this concentration range.

To date, this is the first report on antidiabetic activity of the aerial parts of *M. diffusa* and of the *Minthostachys* genus as well. The results are very interesting and, in particular, the ethyl acetate fraction inhibitory activity against both the tested enzymes, and the *n*-hexane fraction against α-glucosidase activity.

### 3.4. Determination of Anticholinesterase Activity

**Figure 5:**
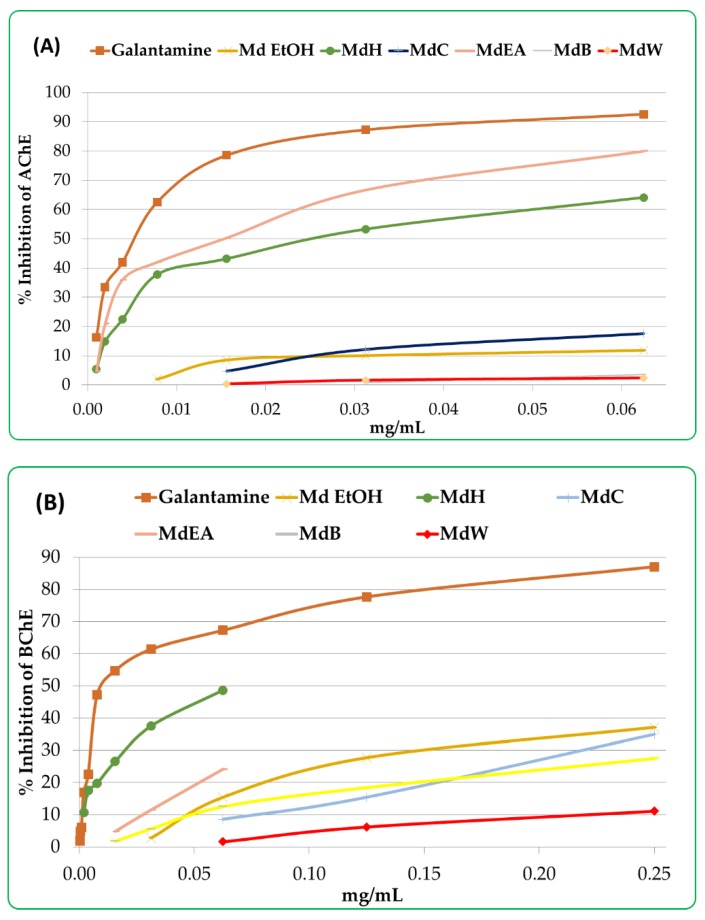
AChE (**A**) and BChE (**B**) inhibition activity of different *M. diffusa* samples. Samples are galantamine, crude ethanol extract (Md EtOH), *n*-hexane fraction (MdH), chloroform fraction (MdC), ethyl acetate fraction (MdEA), *n*-butanol fraction (MdB) and water fraction (MdW).

The inhibitory effects of the *M. diffusa* samples were also tested on acetyl cholinesterase (AChE) and butyryl cholinesterase (BChE) activity and the enzymatic assays demonstrate a concentration-dependent activity (Figure 5).

**Figure 6:**
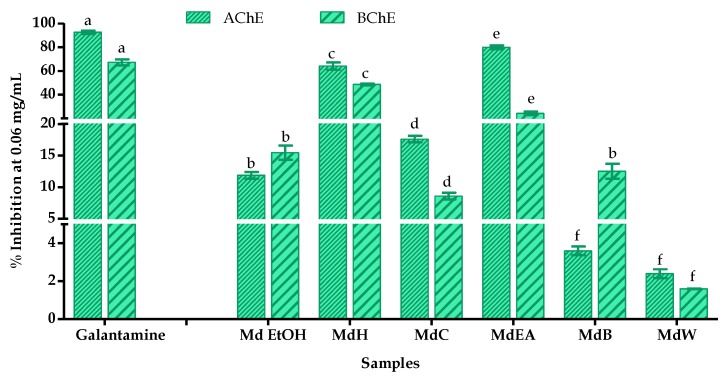
AChE and BChE inhibition in % by *M. diffusa* samples and the positive control galantamine expressed as percentage of inhibition at 0.06 mg/mL. Samples are crude ethanol extract (Md EtOH), *n*-hexane fraction (MdH), chloroform fraction (MdC), ethyl acetate fraction (MdEA), *n*-butanol fraction (MdB) and water fraction (MdW). Data are expressed as mean ± standard deviation from three experiments. In each test, the values with the same letter (a, b, c, d, e, f) are not significant different at the *p* > 0.05 level, 95% confidence limit, according to one-way analysis of variance (ANOVA).

In order to assess the anticholinesterase activities, the results were expressed as percentage of inhibition at a normalized concentration of each sample and the positive control (galantamine) to 0.06 mg/mL (Figure 6). In doing so, the inhibition of AChE from all samples of *M. diffusa* was lower than that of galantamine (92.61 ± 1.41%). Amongst the samples, the MdEA and MdH fractions presented highest AChE inhibitions of 80.00 ± 1.49 and 64.11 ± 3.11%, respectively. A similar trend was observed in the BChE assays, where the MdH and MdEA fractions inhibited 48.65 ± 0.82 and 24.20 ± 1.57%, respectively that were lower compared to galantamine (67.26 ± 2.61%).

This is also the first report on anticholinesterase effects of *M. diffusa* extracts. To date, only the *M. verticillata* of *Minthostachys* genus have been tested for anticholinesterase potential [30]. In a similar approach to our experimental design, the ethanolic extract of *M. verticillata* was partitioned in CH_2_Cl_2_:H_2_O to obtain an organic and an aqueous fraction. Both showed poor AChE inhibitions (≤ 5% at 1 mg/mL). However, other species, such as the *Salvia* genus, belonging to the Lamiaceae family has been reported to possess good cholinesterase inhibition activities [31,32].

### 3.5. Identification and Quantification of Phytochemicals by Liquid Chromatography-Mass Spectrometry

The ethyl acetate fraction of *M. diffusa* (MdEA) was chosen for further LC-MS/MS characterization based on its highest RACI and, also, for its highest antidiabetic and anticholinesterase capacities. More than 30 compounds were detected and some of the polyphenols were (tentatively) identified, through accurate mass measurements, fragmentation pattern and aided by the existing literature, for the first time in *M. diffusa* and in general in the *Minthostachys* genus (Table 3). In the past, only the essential oil of other species belonging to *Minthostachys* genus had been profiled for its phytochemicals that showed mainly monoterpenes [33,34]. Fourteen compounds were identified in the MdEA fraction by comparing their retention times with those of the available commercial standards and subsequently quantified. *M. diffusa* predominantly contained flavonols (quercetin-3,4′-di-glucoside, rutin, quercetin-3-*O*-glucoside, quercetin-3-*O*-arabinoside, kaempferol-3-*O*-glucoside, and quercetin), flavones (apigenin-7-*O*-glycoside, luteolin-rutinoside), flavanones (hesperidin, and naringenin-7-*O*-glucoside), cinnamic acid derivatives (rosmarinic acid 4-coumaric acid and caffeic acid), and triterpenes (corosolic acid, betulinic acid, oleanolic acid and their derivatives).

The most abundant was rosmarinic acid (69.64 ± 1.53 mg/g DW) followed by the quercetin-3-*O*-glucoside (22.87 ± 0.25 mg/g DW). These phenolic compounds have been known for their antioxidant properties. Flavonoids such as luteolin and luteolin-glycosides have been reported to be extremely active with anti-inflammatory and antioxidant properties [35,36].

Triterpene and in particular, maslinic acid, betulinic acid, oleanolic acid as well ursolic acid have also been linked with several biological activities including antioxidant, cholinesterase and α-glucosidase inhibitions [37,38], which can explain the positive activity of the MdEA and MdH fractions. A vast majority of the tentatively identified compounds in Table 3 belonged to triterpenes, which were also present in the MdH fraction.

### 3.6. Potential Anticholinesterase Activity of Identified Triterpenes from MdEA and MdH Fractions

The phytochemical investigation of MdEA and MdH fractions led to the tentative identification of five triterpenes, in particular betulinic, corosolic and oleanolic acids in the MdEA fraction and betulinic, maslinic, oleanolic and ursolic acids in the MdH fraction.

**Figure 7:**
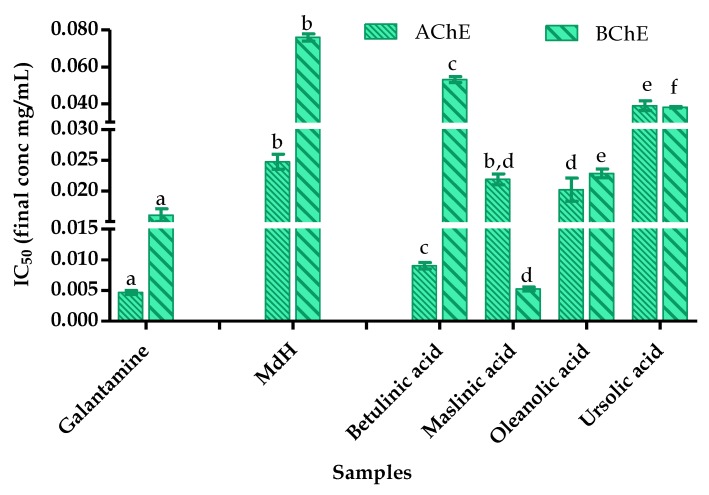
AChE and BChE inhibition by galantamine and *n*-hexane fraction of *M. diffusa* and identified terpenes expressed as IC_50_ values in mg/mL. In each test, the values with the same letter (a, b, c, d, e, f) are not significant different at the *p* > 0.05 level, 95% confidence limit, according to one-way analysis of variance (ANOVA).

Terpenoids are reported as potential leads for the development of cholinesterase inhibitors [39,40,41]. For this reason, in vitro cholinesterase inhibition assays were also performed on the commercially available triterpenes betulinic, corosolic, maslinic, oleanolic and ursolic acids that were found in *M. diffusa*, in order to compare the activities of sample fractions containing these triterpenes. The results expressed as IC_50_ in mg/mL were compared with that of galantamine. All test compounds inhibited the AChE enzyme lesser than the galantamine, where the betulinic, maslinic and oleanolic acids showed IC_50_ of 0.009 ± 0.001 mg/mL, 0.022 ± 0.001 mg/mL and 0.020 ± 0.002 mg/mL, respectively. However, these triterpenes, on its own, were more active than the MdH fraction that contained all these triterpenes (IC_50_ of 0.025 ± 0.001 mg/mL, Figure 7). In BChE assay, only the maslinic acid (IC_50_ of 0.005 ± 0.001 mg/mL) inhibited the enzyme more effectively than the galantamine (IC_50_ of 0.016 ± 0.001 mg/mL). Nonetheless, all tested triterpenes inhibited the BChE enzyme more actively than the MdH fraction.

On the other hand, among all identified triterpenes from the MdEA fraction, only betulinic acid was more effective than the MdEA fraction (IC_50_ of 0.012 ± 0.001 mg/mL) in AChE inhibition. In the BChE assay, the MdEA fraction was not active and also the identified terpenes were less active than galantamine.

Triterpenoids, such as betulinic, corosolic, maslinic, oleanolic, and ursolic acids have been shown to have several biological activities including anticancer, cytotoxic, antitumor, antioxidant, anti-inflammatory, anti-HIV, anti-cholinesterase, α-glucosidase inhibition, antimicrobial, and hepatoprotective activities [37,38,39,40]. It has been demonstrated that betulinic acid also improves learning and memory of aged as well as scopolamine-induced amnesic rats. Furthermore, betulinic acid also decreased lipid peroxidation and nitrite level, and increased the levels of reduced glutathione and superoxide dismutase [40]. Ursolic acid isolated from *Micromeria cilicica* has been shown to inhibit AChE and BChE at IC_50_ of 93.80 and 41.10 μM, respectively [41]. In our assays, the IC_50_ values were similar: 85.35 ± 5.85 μM against AChE and IC_50_ of 83.50 ± 0.94 μM in BChE inhibition.

### 3.7. Molecular Docking of the Identified Terpenes into AChE and BChE

**Figure 8:**
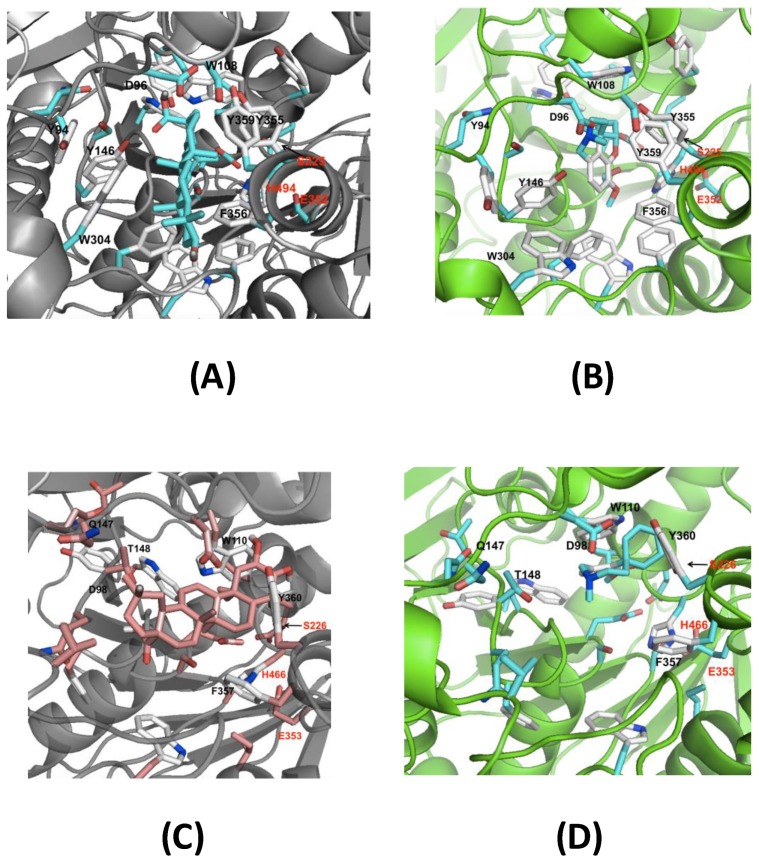
Docking results of betulinic acid (**A**, central cyan moiety) and galantamine (**B**, central cyan/white moiety) in the homology model of AChE; maslinic acid (**C**, central salmon moiety) and galantamine (**D**, central cyan/white moiety) in the homology model of BChE. Residues interacting with the ligands are indicated in black and the residues of the catalytic triad in red.

To get further insight into the inhibitory effects of the *M. diffusa* terpenes on AChE and BChE activity, structural homology models of these enzymes of the in vitro assays were made and used in docking studies. The docking procedure provides the treatment of ligand flexibility within the protein-binding site [42,43]. The results indicated that all terpenes tested had the ability to bind to the active sites of AChE and BChE with eminent affinities (estimated binding energies of −10 to −15 kcal/mol), which is in agreement with a previous study where some AChE-inhibiting compounds (corosolic, oleanolic and ursolic acids) were docked into the active site [42]. Galantamine binds close to the catalytic triad of AChE and BChE, whereas betulinic acid and maslinic acid, which were the most were the most active compounds in AChE and BChE assays, occupy almost the whole binding pocket surfaced with interacting aromatic residues in the corresponding enzyme (Figure 8). Thus, these results demonstrate that all of the terpenes have the potential of binding the active sites of both enzymes and provide valuable insights into the interactions of the inhibitors. The interesting inhibitory activity obtained from in vitro assays of terpenoids identified for the first time in *M. diffusa* suggests that follow-up studies of these compounds are warranted.

## 4. Conclusions

The relative antioxidant capacity index (RACI) evidenced the ethyl acetate (MdEA) fraction of *M. diffusa* as the most active of the five different fractions and of the ethanol crude extract, which also showed the highest inhibition effects against α-amylase, α-glucosidase and cholinesterases. These health-promoting bioactivities may be attributed to the high content of polyphenols, in particular flavonoids and triterpenes. Molecular docking studies on individual authentic triterpenes have confirmed the anticholinesterase actions. The findings provide scientific explanation for the traditional uses of this specie and ascertain the health benefits of the infusions of *M. diffusa* commonly consumed by the local populations. These results also demonstrate that the *M. diffusa* represents a rich source of natural agents for nutraceuticals, food preservatives, functional foods, and cosmetics.

## Figures and Tables

**Table 1:** Results of 2,2′-azino-bis(3-ethylbenzothiazoline-6-sulfonic acid) (ABTS), 2,2-diphenyl-1-picrylhydrazyl (DPPH) and Super Oxide anion (SO) scavenging activity, Ferric Reducing Antioxidant Power (FRAP) and β-Carotene Bleaching (BCB) of *M. diffusa* samples.

Samples	ABTS (mgTE/g)	DPPH (mgTE/g)	SO IC_25_ (mg/mL)	FRAP (mgTE/g)	BCB %AA
**Md EtOH**	146.07 ± 4.20 ^a^	113.05 ± 3.51 ^a^	0.92 ± 0.08 ^a^	224.67 ± 3.37 ^a^	10.12 ± 0.52 ^a^
**MdH**	nc	nc	nc	27.27 ± 0.99 ^b^	11.01 ± 0.33 ^a^
**MdC**	29.61 ± 0.74 ^b^	17.64 ± 0.03 ^b^	1.03 ± 0.08 ^a,b^	22.18 ± 0.90 ^b^	13.03 ± 0.49 ^b^
**MdEA**	444.76 ± 28.24 ^c^	281.64 ± 7.93 ^c^	0.62 ± 0.04 ^c^	574.86 ± 9.14 ^c^	19.13 ± 0.93 ^c^
**MdB**	218.56 ± 9.38 ^d^	122.30 ± 2.77 ^a^	0.26 ± 0.01 ^d^	297.75 ± 5.71 ^d^	nc
**MdW**	45.85 ± 0.42 ^b^	37.01 ± 1.63 ^d^	1.14 ± 0.09 ^b^	64.67 ± 1.63 ^e^	nc

Samples are crude ethanol extract (Md EtOH), *n*-hexane fraction (MdH), chloroform fraction (MdC), ethyl acetate fraction (MdEA), *n*-butanol fraction (MdB) and water fraction (MdW). Data are expressed as means ± standard deviation from three experiments. Different superscripts in the same row (a, b, c, d, e) indicate significant difference (*p* < 0.05); nc = not calculable.

**Table 2:** Pearson correlation coefficient calculated among tested *M. diffusa* samples.

	TPC	TTeC	ABTS	DPPH	SO	NO	FRAP	BCB
**TPC**	1.00							
**TTeC**	−0.26	1.00						
**ABTS**	0.96	−0.31	1.00					
**DPPH**	0.94	−0.23	0.99	1.00				
**SO**	0.69	−0.40	0.50	0.44	1.00			
**NO**	0.44	−0.31	0.21	0.13	0.91	1.00		
**FRAP**	0.96	−0.24	1.00	0.99	0.50	0.22	1.00	
**BCB**	0.26	0.06	0.40	0.43	−0.40	−0.58	0.38	1.00

Total Polyphenolic Content (TPC), Total Terpenoid Content (TTeC), ABTS assay, DPPH assay, Super Oxide anion scavenging activity (SO), Nitric Oxide radical scavenging activity (NO), Ferric Reducing Antioxidant Power assay (FRAP) and β-Carotene Bleaching assay (BCB).

**Table 3:** Characterisation of phytochemicals present in ethyl acetate fraction of *M. diffusa* by liquid chromatography-tandem mass spectrometry. Quantities of the detected compounds were determined using commercial standards (in bolds); nq = not quantified.

Pk. no.	RT (min)	[M-H]^−^*m*/*z* Observed	[M-H]^−^*m*/*z* Calculated	Predicted Molecular Formula	MS/MS (*m*/*z*)	Compound Identity	mg/g DW
**1**	6.18	179.0367	179.0344	C_9_H_8_O_4_	135, 79	**Caffeic acid**	2.49 ± 0.01
2	6.84	461.1769	461.1753	C_31_H_26_O_4_	329, 301	unknown	nq
**3**	6.71	625.1441	625.1405	C_27_H_30_O_17_	343, 301, 271, 255, 179, 151	**Quercetin-3,4′-di-glucoside**	0.03 ± 0.02
**4**	7.17	609.1473	609.1456	C_27_H_30_O_16_	300, 285, 271, 255, 179, 151	**Rutin**	1.63 ± 0.07
**5**	7.37	463.0876	463.0877	C_21_H_20_O_12_	300, 271, 255, 179, 151	**Quercetin-3-*O*-glucoside**	22.87 ± 0.25
6	7.67	505.0982	505.098	C_23_H_22_O_13_	300, 271, 255, 179, 161, 151	Quercetin derivative	Nq
**7**	7.70	433.0742	433.0771	C_20_H_18_O_11_	300, 271, 255, 179,151	**Quercetin-3-*O*-arabinoside**	1.92 ± 0.02
**8**	7.87	447.0921	447.0927	C_21_H_20_O_11_	284, 255, 227	**Kaempferol-3-*O*-glucoside**	2.07± 0.15
**9**	8.00	609.1848	609.1819	C_28_H_34_O_15_	325, 301, 286, 242, 199, 164, 125	**Hesperidin**	1.10 ± 0.09
**10**		431.0981	431.0978	C_21_H_20_O_10_	269, 239, 224	**Apigenin-7-*O*-glycoside**	0.54 ± 0.05
**11**		433.1125	433.1135	C_21_H_22_O_10_	151, 107	**Naringenin-7-*O*-glucoside**	0.17 ± 0.01
**12**	8.24	359.0766	359.0767	C_18_H_16_O_8_	197, 179, 161, 135, 133, 123, 73	**Rosmarinic acid**	69.64 ± 1.53
13	8.73	609.1457	609.1456	C_27_H_30_O_16_	463, 323, 300, 285, 271, 255, 179, 161, 151	Quercetin-*O*-glucoside- rhamnoside	nq
14	8.79	533.1882	533.1870	C_23_H_34_O_14_	387, 374, 207, 163, 145, 119, 101	Coumaric acid derivative	nq
15		471.1216	471.1232	C_31_H_20_O_5_	307, 205, 163, 145, 119, 101	Coumaric acid derivative	nq
**16**		163.0392	163.0395	C_9_H_8_O_3_	119, 93	**4-Coumaric acid**	0.23 ± 0.02
17	9.15	593.1697	593.1506	C_27_H_30_O_15_	327, 309, 285, 270, 241, 164, 151	Luteolin-rutinoside	nq
**18**		285.0384	285.0399	C_15_H_10_O_6_	151, 133	**Luteolin**	1.00 ± 0.00
**19**	9.39	301.0362	301.0348	C_15_H_10_O_7_	179, 151	**Quercetin**	2.07 ± 0.09
20	9.69	373.0908	373.0923	C_19_H_18_O_8_	197, 179, 161, 135, 117, 107	Rosmarinic acid methyl ester	nq
21	11.34	487.3447	487.3424	C_30_H_48_O_5_	469, 441, 405, 397,389, 85, 73	Asiatic acid type	nq
22	11.50	487.3447	487.3424	C_30_H_48_O_5_	469, 441, 405, 397,389, 85, 73	Asiatic acid type	nq
23	12.03	829.4156	829.4163	C_48_H_62_O_12_	811, 789, 667, 649, 553, 359, 179, 161, 135	Rosmarinic acid derivative	nq
24	12.82	471.3475	471.3474	C_30_H_48_O_4_	427, 425, 409, 353, 337, 57	Corosolic type triterpenoid	nq
25	13.81	501.3549	501.3580	C_31_H_50_O_5_	469, 421, 407, 389	Asiatic acid methyl ester	nq
26	14.18	813.4201	813.4214	C_48_H_62_O_11_	651, 453, 359, 197, 179, 161, 135, 73	Rosmarinic acid derivative	nq
27	14.51	455.3527	455.3525	C_30_H_48_O_3_	411, 393, 381, 351, 83, 71, 57	Oleanolic type triterpenoid	nq
28	15.22	469.3342	469.3318	C_30_H_46_O_4_	451, 425, 421, 407, 391, 377, 353, 337, 137	Corosolic type triterpenoid	nq
29	15.67	471.3453	471.3474	C_30_H_48_O_4_	453, 411, 353, 337, 121, 113, 97, 71, 57	Corosolic type triterpenoid	nq
30	16.38	469.3342	469.3318	C_30_H_46_O_4_	451, 425, 421, 407, 391, 377, 353, 337, 137	Corosolic type triterpenoid	nq
31	17.04	469.3342	469.3318	C_30_H_46_O_4_	451, 425, 421, 407, 391, 377, 353, 337, 137	Corosolic type triterpenoid	nq
**32**	17.41	471.3453	471.3474	C_30_H_48_O_4_	453, 411, 353, 337, 121, 113, 97, 71, 57	**Corosolic acid**	4.06 ± 2.46
33	20.18	453.3460	453.3369	C_30_H_46_O_3_	405, 391, 389, 371, 337, 97	Oleanolic type triterpenoid	nq
**34**	20.84	455.3521	455.3525	C_30_H_48_O_3_	452, 407, 391, 389, 375, 373, 189, 183, 137	**Betulinic acid**	3.93 ± 0.63
**35**	21.33	455.3539	455.3525	C_30_H_48_O_3_	407, 391, 389, 375, 373, 189, 183, 137, 97	**Oleanolic acid**	7.26 ± 1.56

## References

[1] Buchanan B.B., Gruissem W., Jones R.L. (2000). Biochemistry & Molecular Biology of Plants.

[2] Bourgaud F., Gravot A., Milesi S., Gontier E. (2001). Production of plant secondary metabolites: A historical perspective. Plant Sci..

[3] Pinakin D.J., Kumar V., Suri S., Sharma R., Kaushal M. (2020). Nutraceutical potential of tree flowers: A comprehensive review on biochemical profile, health benefits, and utilization. Food Res. Int..

[4] Tang G.-Y., Meng X., Gan R.-Y., Zhao C.-N., Liu Q., Feng Y.-B., Li S., Wei X.-L., Atanasov A.G., Corke H. (2019). Health functions and related molecular mechanisms of tea components: An update review. Int. J. Mol. Sci..

[5] Abdalla M.A., Zidorn C. (2020). The genus *Tragopogon* (asteraceae): A review of its traditional uses, phytochemistry, and pharmacological properties. J. Ethnopharmacol..

[6] Schmidt-Lebuhn A.N. (2008). Ethnobotany, biochemistry and pharmacology of *Minthostachys* (Lamiaceae). J. Ethnopharmacol..

[7] Lock O., Perez E., Villar M., Flores D., Rojas R. (2016). Bioactive compounds from plants used in Peruvian traditional medicine. Nat. Prod. Commun..

[8] Cariddi L., Escobar F., Moser M., Panero A., Alaniz F., Zygadlo J., Sabini L., Maldonado A. (2011). Monoterpenes isolated from *Minthostachys verticillata* (Griseb.) Epling essential oil modulates immediate-type hypersensitivity responses in vitro and *in vivo*. Planta Med..

[9] Montironi I.D., Cariddi L.N., Reinoso E.B. (2016). Evaluation of the antimicrobial efficacy of *Minthostachys verticillata* essential oil and limonene against *Streptococcus uberis* strains isolated from bovine mastitis. Rev. Argent. Microbiol..

[10] Mora F.D., Araque M., Rojas L.B., Ramirez R., Silva B., Usubillaga A. (2009). Chemical composition and in vitro antibacterial activity of the essential oil of *Minthostachys mollis* (Kunth) Griseb Vaught from the Venezuelan Andes. Nat. Prod. Commun..

[11] Cantín Á., Lull C., Primo J., Miranda M.A., Primo-Yúfera E. (2001). Isolation, structural assignment and insecticidal activity of (−)-(1S,2R,3R,4S)-1,2-Epoxy-1-methyl-4-(1-methylethyl)-cyclohex-3-yl acetate, a natural product from *Minthostachys tomentosa*. Tetrahedron Asymmetry.

[12] Roberts R.O., Knopman D.S., Przybelski S.A., Mielke M.M., Kantarci K., Preboske G.M., Senjem M.L., Pankratz V.S., Geda Y.E., Boeve B.F. (2014). Association of type 2 diabetes with brain atrophy and cognitive impairment. Neurology.

[13] Aguirre-Acevedo D.C., Lopera F., Henao E., Tirado V., Muñoz C., Giraldo M., Bangdiwala S.I., Reiman E.M., Tariot P.N., Langbaum J.B. (2016). Cognitive decline in a colombian kindred with autosomal dominant Alzheimer disease: A retrospective cohort study. JAMA Neurol..

[14] Redondo M.T., Beltrán-Brotóns J.L., Reales J.M., Ballesteros S. (2016). Executive functions in patients with Alzheimer’s disease, type 2 diabetes mellitus patients and cognitively healthy older adults. Exp. Gerontol..

[15] Faraone I., Rai D., Chiummiento L., Fernandez E., Choudhary A., Prinzo F., Milella L. (2018). Antioxidant activity and phytochemical characterization of *Senecio clivicolus* Wedd. Molecules.

[16] Lamorte D., Faraone I., Laurenzana I., Milella L., Trino S., De Luca L., Del Vecchio L., Armentano M., Sinisgalli C., Chiummiento L. (2018). Future in the past: *Azorella glabra* Wedd. as a source of new natural compounds with antiproliferative and cytotoxic activity on Multiple Myeloma cells. Int. J. Mol. Sci..

[17] Faraone I., Rai D.K., Russo D., Chiummiento L., Fernandez E., Choudhary A., Milella L. (2019). Antioxidant, Aantidiabetic, and Aanticholinesterase Aactivities and Pphytochemical Pprofile of *Azorella glabra* Wedd. Plants.

[18] Singleton V.L., Orthofer R., Lamuela-Raventós R.M. (1999). Analysis of total phenols and other oxidation substrates and antioxidants by means of Folin-Ciocalteu reagent. Methods Enzymol..

[19] Lin J.-Y., Tang C.-Y. (2007). Determination of total phenolic and flavonoid contents in selected fruits and vegetables, as well as their stimulatory effects on mouse splenocyte proliferation. Food Chem..

[20] Chakraborty S., Guchhait S., Saha S., Biswas S. (2012). Estimation of total terpenoids concentration in plant tissues using a monoterpene, linalool as standard reagent. Protoc. Exch..

[21] Re R., Pellegrini N., Proteggente A., Pannala A., Yang M., Rice-Evans C. (1999). Antioxidant activity applying an improved ABTS radical cation decolorization assay. Free Radic. Biol. Med..

[22] Stratil P., Klejdus B., Kubáň V. (2006). Determination of total content of phenolic compounds and their antioxidant activity in vegetables evaluation of spectrophotometric methods. J. Agric. Food Chem..

[23] Fidelis Q.C., Faraone I., Russo D., Aragão Catunda F.E., Vignola L., de Carvalho M.G., De Tommasi N., Milella L. (2019). Chemical and biological insights of *Ouratea hexasperma* (A. St.-Hil.) Baill.: A source of bioactive compounds with multifunctional properties. Nat. Prod. Res..

[24] Waterhouse A., Bertoni M., Bienert S., Studer G., Tauriello G., Gumienny R., Heer F.T., de Beer T.A.P., Rempfer C., Bordoli L. (2018). SWISS-MODEL: Homology modelling of protein structures and complexes. Nucleic Acids Res..

[25] Krieger E., Joo K., Lee J., Lee J., Raman S., Thompson J., Tyka M., Baker D., Karplus K. (2009). Improving physical realism, stereochemistry, and side-chain accuracy in homology modeling: Four approaches that performed well in CASP8. Proteins Struct. Funct. Bioinform..

[26] Trott O., Olson A.J. (2010). AutoDock Vina: Improving the speed and accuracy of docking with a new scoring function, efficient optimization, and multithreading. J. Comput. Chem..

[27] Palacios S.M., del Corral S., Carpinella M.C., Ruiz G. (2010). Screening for natural inhibitors of germination and seedling growth in native plants from Central Argentina. Ind. Crops Prod..

[28] Vaquero M.R., Serravalle L.T., De Nadra M.M., De Saad A.S. (2010). Antioxidant capacity and antibacterial activity of phenolic compounds from Argentinean herbs infusions. Food Control.

[29] Solis-Quispe L., Tomaylla-Cruz C., Callo-Choquelvica Y., Solís-Quispe A., Rodeiro I., Hernández I., Fernández M.D., Pino J.A. (2016). Chemical composition, antioxidant and antiproliferative activities of essential oil from *Schinus areira* L. and *Minthostachys spicata* (Benth.) Epl. grown in Cuzco, Peru. J. Essent. Oil Res..

[30] Carpinella M.C., Andrione D.G., Ruiz G., Palacios S.M. (2010). Screening for acetylcholinesterase inhibitory activity in plant extracts from Argentina. Phytother. Res..

[31] Savelev S.U., Okello E.J., Perry E.K. (2004). Butyryl-and acetyl-cholinesterase inhibitory activities in essential oils of *Salvia* species and their constituents. Phytother. Res..

[32] Mukherjee P.K., Kumar V., Mal M., Houghton P.J. (2007). Acetylcholinesterase inhibitors from plants. Phytomedicine.

[33] Alkire B.H., Tucker A.O., Maciarello M.J. (1994). Tipo, *Minthostachys mollis* (lamiaceae): An Ecuadorian mint. Econ. Bot..

[34] Senatore F. (1995). Composition of the essential oil of *Minthostachys spicata* (Benth.) Epl. Flavour Fragr. J..

[35] López-Lázaro M. (2009). Distribution and biological activities of the flavonoid luteolin. Mini Rev. Med. Chem..

[36] Uma Devi P., Ganasoundari A., Vrinda B., Srinivasan K., Unnikrishnan M. (2000). Radiation protection by the *Ocimum* flavonoids orientin and vicenin: Mechanisms of action. Radiat. Res..

[37] Leal A.S.M. (2012). Preparation and biological evaluation of new triterpene derivates of ursolic and oleanolic acid. Ph.D. Thesis.

[38] Sultana N. (2011). Clinically useful anticancer, antitumor, and antiwrinkle agent, ursolic acid and related derivatives as medicinally important natural product. J. Enzym. Inhib. Med. Chem..

[39] Jamila N., Khairuddean M., Yeong K.K., Osman H., urugaiyah V. (2015). Cholinesterase inhibitory triterpenoids from the bark of *Garcinia hombroniana*. J. Enzym. Inhib. Med. Chem..

[40] Ruhal P., Dhingra D. (2018). Ameliorative effect of betulinic acid on ageing and scopolamine-induced learning and memory impairment in rats. Asian J. Pharm. Pharmacol..

[41] Öztürk M., Kolak U., Topçu G., Öksüz S., Choudhary M.I. (2011). Antioxidant and anticholinesterase active constituents from *Micromeria cilicica* by radical-scavenging activity-guided fractionation. Food Chem..

[42] Bahadori M.B., Dinparast L., Valizadeh H., Farimani M.M., Ebrahimi S.N. (2016). Bioactive constituents from roots of *Salvia syriaca* L.: Acetylcholinesterase inhibitory activity and molecular docking studies. South Afr. J. Bot..

[43] Geromichalos G.D., Lamari F.N., Papandreou M.A., Trafalis D.T., Margarity M., Papageorgiou A., Sinakos Z. (2012). Saffron as a source of novel acetylcholinesterase inhibitors: Molecular docking and in vitro enzymatic studies. J. Agric. Food Chem..

